# CT-based radiomics for prediction of therapeutic response to Everolimus in metastatic neuroendocrine tumors

**DOI:** 10.1007/s11547-022-01506-4

**Published:** 2022-06-18

**Authors:** Damiano Caruso, Michela Polici, Maria Rinzivillo, Marta Zerunian, Ilaria Nacci, Matteo Marasco, Ludovica Magi, Mariarita Tarallo, Simona Gargiulo, Elsa Iannicelli, Bruno Annibale, Andrea Laghi, Francesco Panzuto

**Affiliations:** 1 Department of Medical Surgical Sciences and Translational Medicine, “Sapienza”-University of Rome, Sant′Andrea University Hospital, AOU Sant’Andrea, Via di Grottarossa, 1035-1039, 00189 Rome, Italy; 2 Radiology Unit, Sant′Andrea University Hospital, AOU Sant’Andrea, 00189 Rome, Italy; 3 Digestive Disease Unit, Sant′Andrea University Hospital, AOU Sant’Andrea, 00189 Rome, Italy; 4 ENETS Center of Excellence of Rome, Sant′Andrea University Hospital, AOU Sant′Andrea, 00189 Rome, Italy; 5 Department of Surgery “Pietro Valdoni”, Sapienza University of Rome, 00161 Rome, Italy

**Keywords:** Radiomics, Neuroendocrine tumors, Everolimus, Response to treatment

## Abstract

**Aim:**

To test radiomic approach in patients with metastatic neuroendocrine tumors (NETs) treated with Everolimus, with the aim to predict progression-free survival (PFS) and death.

**Materials and methods:**

Twenty-five patients with metastatic neuroendocrine tumors, 15/25 pancreatic (60%), 9/25 ileal (36%), 1/25 lung (4%), were retrospectively enrolled between August 2013 and December 2020. All patients underwent contrast-enhanced CT before starting Everolimus, histological diagnosis, tumor grading, PFS, overall survival (OS), death, and clinical data collected. Population was divided into two groups: responders (PFS ≤ 11 months) and non-responders (PFS > 11 months). 3D segmentation was performed on whole liver of naïve CT scans in arterial and venous phases, using a dedicated software (3DSlicer v4.10.2). A total of 107 radiomic features were extracted and compared between two groups (T test or Mann–Whitney), radiomics performance assessed with receiver operating characteristic curve, Kaplan–Meyer curves used for survival analysis, univariate and multivariate logistic regression performed to predict death, and interobserver variability assessed. All significant radiomic comparisons were validated by using a synthetic external cohort. *P* < 0.05 is considered significant.

**Results:**

15/25 patients were classified as responders (median PFS 25 months and OS 29 months) and 10/25 as non-responders (median PFS 4.5 months and OS 23 months). Among radiomic parameters, *Correlation* and *Imc1* showed significant differences between two groups (*P* < 0.05) with the best performance (internal cohort AUC 0.86–0.84, P < 0.0001; external cohort AUC 0.84–0.90; *P* < *0.0001*). *Correlation* < 0.21 resulted correlated with death at Kaplan–Meyer analysis (*P* = *0.02*). Univariate analysis showed three radiomic features independently correlated with death, and in multivariate analysis radiomic model showed good performance with AUC 0.87, sensitivity 100%, and specificity 66.7%. Three features achieved 0.77 ≤ ICC < 0.83 and one ICC = 0.92.

**Conclusions:**

In patients affected by metastatic NETs eligible for Everolimus treatment, radiomics could be used as imaging biomarker able to predict PFS and death.

## Introduction

Neuroendocrine neoplasms (NENs) are rare and indolent tumors, arising from diffuse neuroendocrine cells and the most common neoplasms occur in the gastroenteropancreatic tract (GEP-NETs) and in the lung [1]. An updating of WHO classification divided NENs in well-differentiated neuroendocrine tumors (NETs) and poorly differentiated neuroendocrine carcinomas (NECs) according to differentiation degree and morphological features, in order to classify the more aggressive neoplasms as carcinomas [2].

In the new era of target therapy, Everolimus has been used in the treatment of advanced NETs, in management of advanced progressive disease [3, 4]. One of the main clinical challenges is to predict efficacy of Everolimus before starting the therapy, by stratifying patients as responders and non-responders, then predicting progression-free survival (PFS) and death. In fact, both clinical and histological data (e.g., tumor grade, age, sex, and prior chemotherapy) resulted to have limited accuracy to predict PFS and death with consistent and reproducible results [4, 5].

In such scenario, radiomics could be an emerging noninvasive biomarker, having the expectancy to predict patient prognosis on the basis of liver microarchitecture, microenvironmental, and heterogeneity by extracting radiomic features from volumetric liver segmentation [6–9]. NENs radiomic approach was studied and tested by several groups, with a specific focus on tumor degree differentiation and differential diagnosis between neuroendocrine and non-neuroendocrine neoplasms [10–16]. The major results were reached in tumor degree differentiation with the goal to overcome and support conventional tumor biopsy, often altered by substantial intrinsic bias (e.g., lesion sampling, operator experience, and bleeding) [17]. In the future landscape of personalized medicine, radiomics might enter in the structured workflow of NENs by providing several objective parameters useful for stratifying patients according to tumor aggressiveness, risk of recurrence, and mortality [6, 7]. In this study we proposed both a comparison and survival analysis between responders and non-responder NETs to Everolimus, then we built a radiomic model having as clinical endpoint the death. The goal of the study was to provide an imaging tool to screen patients with high risk of aggressive disease before starting the therapy.

On the best of our knowledge, there are no studies which tested radiomics performance to predict prognosis, in terms of PFS and death, in patients with NET before starting Everolimus. The aim of this study is to investigate the performance of radiomic approach, by analyzing naïve CT scans, in predicting PFS and to test any correlations with death in patients affected by metastatic NETs suitable for Everolimus treatment.

## Materials and methods

### Patient selection

**Fig. 1:**
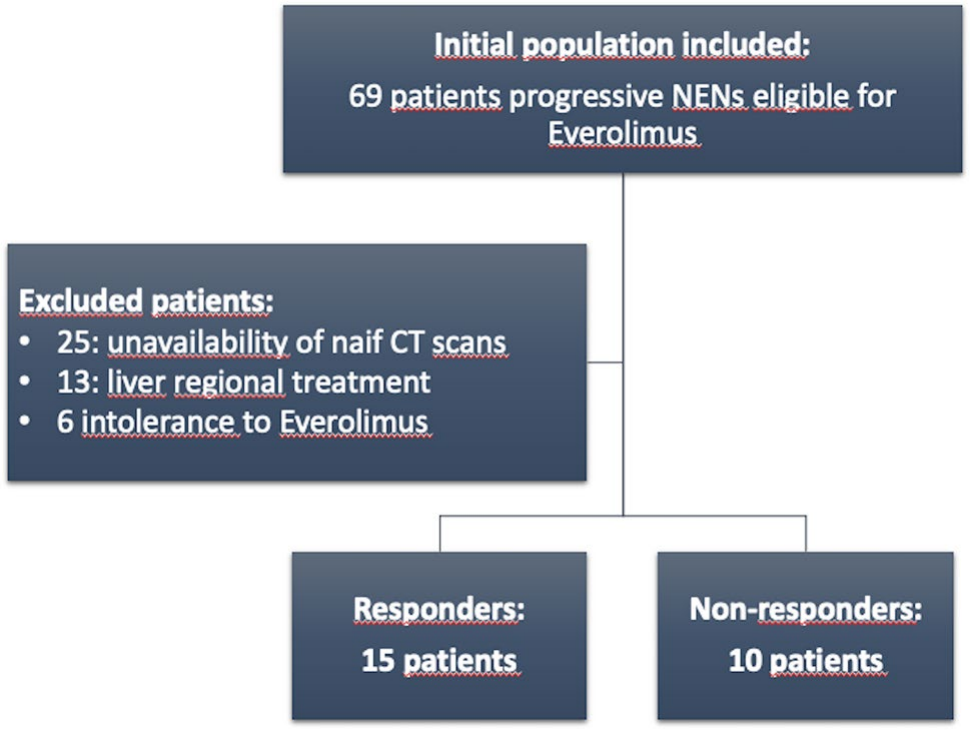
Patients enrollment flowchart

This retrospective observational study was in accordance with the Declaration of Helsinki. All procedures were approved by the ENETS Center of Excellence of Rome (Sant’Andrea University Hospital)  Institutional Review Board. It was structured by selecting all patients affected by NETs with liver metastases, afferents at the ENETS Center of Excellence of Rome (Sant'Andres University Hospital), from August 2013 to December 2020, eligible to be treated with Everolimus (well-differentiated gastroenteropancreatic or lung NENs with documented progressive disease) [4]. All participants provided the informed consensus, the approval of Institutional Review Board was obtained. Epidemiological and clinical data were collected for each patients including sex, age, tumor grading, Ki67, overall survival (OS), and PFS. Patients were selected according to the following inclusion criteria: (a) patients with histological diagnosis of NETs, (b) evidence of liver metastases, (c) availability of clinical data, and d) availability of contrast-enhanced naïve CT scans. Exclusion criteria were the following: (a) intolerance of Everolimus and (b) patients with previous liver surgery or locoregional treatment. PFS and OS were evaluated from the time of beginning of Everolimus treatment. From an initial population of 69 patients, 25 patients with progressive metastatic NETs were enrolled and divided into two groups: responders and non-responders according to the PFS ≤ 11 months and PFS > 11 months, respectively (Fig. 1) [18, 19]. A synthetic external validation cohort was built by using the adaptive synthetic sampling approach (ADASYN) [20], achieving 87 synthetic patients for the group of responders and 90 for the non-responders.

### CT acquisition protocol

Patients with histological diagnosis of metastatic liver disease in NETs, eligible for Everolimus treatment underwent multiphases CT scans before starting Everolimus. CT scans were obtained by using 128-slice CT (GE Revolution EVO Slice CT Scanner, GE Healthcare, Milwaukee, WI, USA), patients were in supine position and the scans were performed in cranio-caudal direction at end-inspiration. Z-axis included the entire abdomen, from the diaphragm to the pubic symphysis for unenhanced, late arterial, portal venous, and delayed phases. In this study were selected for the radiomic analysis late arterial and portal venous phases.

For each patient, the volume of contrast medium (CM) was tailored in accordance with lean body weight (LBW) [21, 22]:

$$\mathrm{CM volume }(\mathrm{mL})=\frac{0.7\mathrm{gI x LBW }(\mathrm{kg})}{\mathrm{CM concentration }(\frac{\mathrm{mgI}}{\mathrm{mL}})}.$$

The administration of contrast medium bolus (iopromide 370 mg I/mL, Ultravist 370; Bayer AG, Berlin, Germany) and the saline solution (45 mL) was performed through contrast media injection system (MEDRAD® Centargo CT Injection System) with a flow rate of 3.5 mL/s by an antecubital venous access (18–20 gauge). The bolus-tracking method (Smart Prep, GE, Milwaukee, WI) was used for the contrast-enhanced CT phases, setting within the abdominal aorta, at level of celiac tripod, a 150 HU-threshold region of interest. All patients were studied with unenhanced, late arterial (18 s from threshold achieved) and portal venous (70 s from threshold achieved). CT scans were obtained by setting the following technical parameters: tube voltage 100 kV; tube current modulation 130-300mAs by using SMART mA (GE Healthcare, Milwaukee, USA); spiral pitch factor 0.98; collimation 64 × 0.625 mm; time of rotation 0.6 s. Standard soft tissue reconstruction, by using Iterative Reconstruction at 40% (ASiR-V, GE Healthcare, Milwaukee, USA), was used for all CT images at slice thickness of 1.25 mm.

### CT scans segmentation analysis

**Fig. 2:**
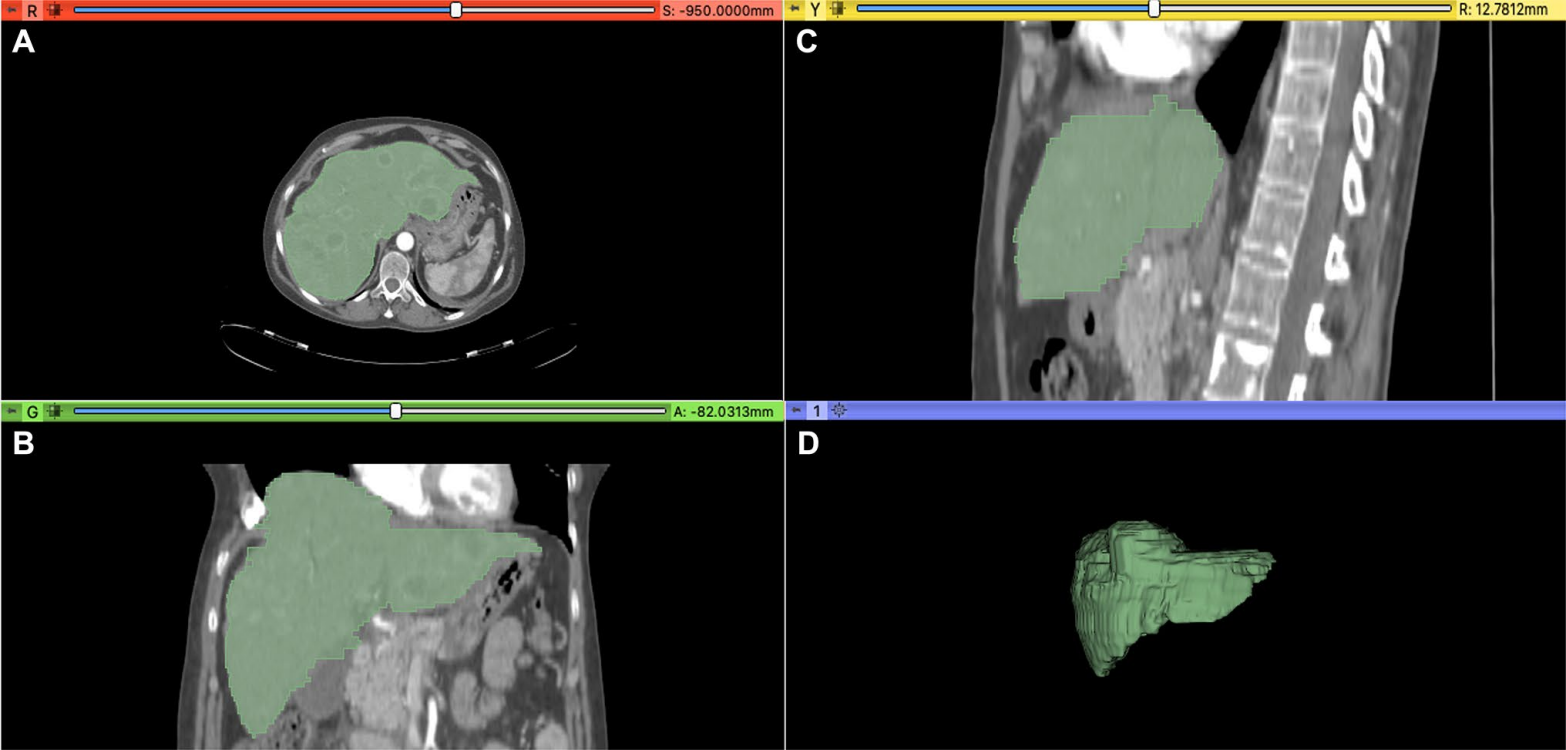
3D manually segmentation of liver parenchyma in arterial phase, performed by using Slicer software (version 4.10.2, http://www.slicer.org), of 54-year-old woman affected by pancreatic NETs (G2) with liver metastases before starting Everolimus treatment. Figure displays axial **A**, Coronal **B**, Sagittal **C**, and 3D Volumetric **D** segmentation of metastatic liver parenchyma

Two expert abdominal radiologists (DC and MZ of 10 and 8 years of experience) independently performed volumetric liver segmentation of all naïve CT scans by using open-source 3D Slicer software (version 4.10.2, http://www.slicer.org) on both late arterial and portal venous phases. Slice-by-slice a volumetric region of interest was manually drawn, with the goal of covering total liver volume and avoiding the liver vessels or the main biliary ducts (Fig. 2). The radiologists have drawn the regions of interest (ROIs) with the same criteria for both arterial and portal phase, to maintain the same ROIs for both phases as much as possible.

### Radiomic features extraction

3D Slicer Radiomics extension (pyradiomics library [23]) was used to extract 107 radiomic features from late arterial and venous phases CT scans, including first- and second-order features: 19 features first-order statistics, 13 features 2D and 3D shape, 16 features gray-level size zone matrix (GLSZM), 5 features neighboring gray-tone difference matrix (NGTDM), 14 features gray-level dependence matrix (GLDM), 24 features gray-level co-occurrence matrix (GLCM), and 16 features gray-level run length matrix (GLRLM).

### Statistical analysis

Continuous parameters were reported as mean ± standard deviation. To compare continuous variables were used Student T test and Mann–Whitney U test based on Gaussian normality or non-normality, respectively. Categorial variables were reported with numbers and percentages, then compared with Fisher’s exact test or χ2 test with or without Yates correction. Performance of significant radiomic features was tested with receiver operating curve (ROC), calculating area under the curve (AUC), sensitivity, specificity, and cutoff values considering PFS (PFS ≤ 11 months and PFS > 11 months) as endpoint. All significant radiomic features were also tested and performance validated through the synthetic external cohort. Kaplan–Meyer survival analysis was applied to test the correlation with death, and log-rank test for *P* values calculating. All clinical and radiomic features were tested with univariate enter logistic regression analysis as predictors of death at naïve CT scan. All features resulted to be significant (*P* < 0.05) were included in multivariable backward logistic regression analysis with the aim to build a radiomic model to predict patients’ death. Statistical significance was assessed with a *P* < 0.05. Statistical analysis was performed with MedCalc (MedCalc Software, version15, Ostend, Belgium). Bonferroni correction was applied to adjust the multiple comparisons. Interobserver variability was also assessed, the features were considered unstable with ICC < 0.75, stable with 0.75 ≤ ICC < 0.9, and excellent with ICC ≥ 0.9.

## Results

### Study population

Population included 25 patients, 11 males and 14 females (44% and 56%, respectively). A sub-analysis according to the primary site, 15 (60%) were pancreatic NETs, 9 (36%) were ileal NETs, and 1 (4%) was lung NET. Concerning tumors grading 5 (20%) were NETs G1, 19 (76%) were NETs G2, and 1 (4%) was NET G3. Population showed a median PFS and OS of 15  and 21 months, respectively. Fifteen patients (60%) resulted to be responders and 10 (40%) were considered non-responders according to PFS. In the analysis of two patient groups, it was showed a median PFS of 27  and 4.5 months in responders and non-responders, respectively (Table 1). Overall, 19 patients died during follow-up, resulting in a mortality rate of 76%.

**Table 1:** Patient clinical data

Patients Characteristics	N patients (n = 25)	%	*P*
*Median age*	60	–	–
Male	11/25	44%	–
Deaths	19/25	76%	–
*Primary*			
Pancreatic	15/25	60%	–
Ileal	9/25	36%	–
Lung	1/25	4%	–
*Grading*			
NET G1	5/25	20%	–
NET G2	19/25	76%	–
NET G3	1/25	4%	–
Overall PFS Median	15 months	–	–
Overall OS Median	21 months	–	–

PFS ≤ 11 (n = 10/25)		PFS >$11$ (n = 15/25)		
PFS Median	4.5 months	PFS Median	25 months	**0.009**
OS Median	13	OS Median	29	0.08
Deaths	8	Deaths	11	

Bold value denotes statistical significance

^*^PFS Progression-free survival, *OS* Overall survival

### 3D segmentation and radiomic features

**Fig. 3:**
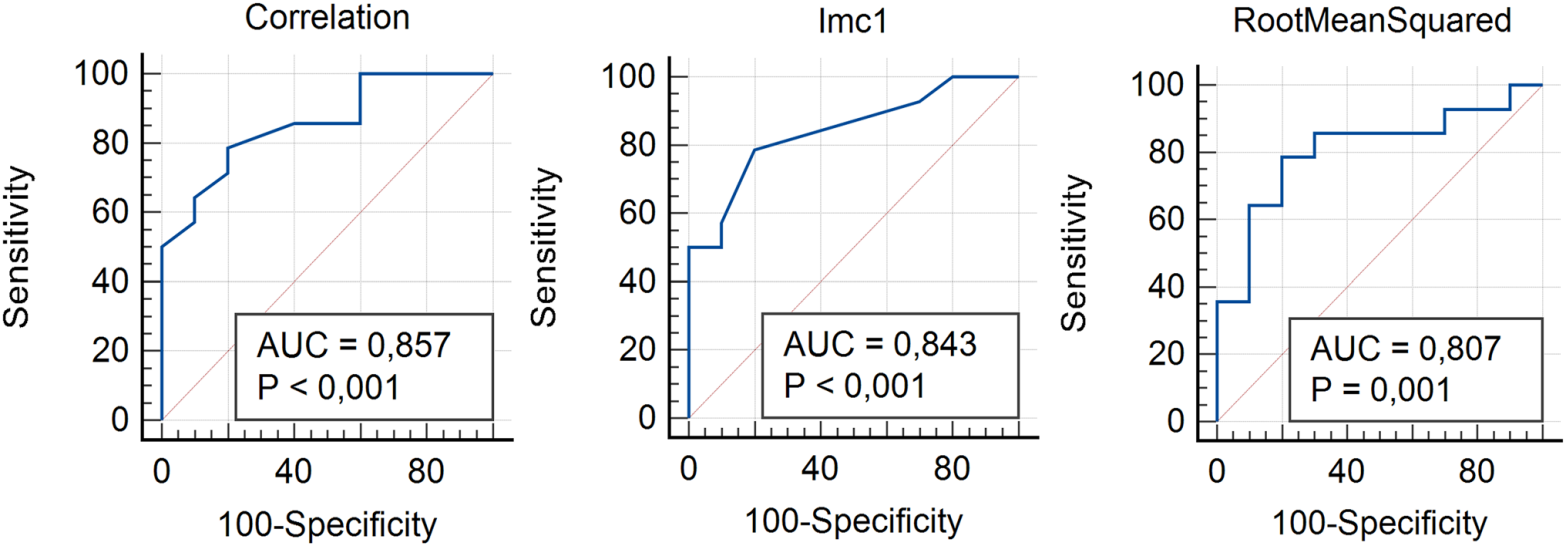
The most performant radiomic features in the comparison between responders and non-responders tested with receiving operative characteristics (ROC) curve in internal cohort. For each curve, P values and area under the curve (AUC) are specified

From the volumetric segmentation of liver parenchyma were extracted 107 radiomic features on both late arterial and portal phases of naïve CT scans. In the comparison between responders and non-responders, ten radiomic parameters resulted to be significantly different (*P* < 0.05) (Table 2). Among radiomic features extracted from arterial phase, four First-Order features (10Percentile, Mean, Median, and RootMeanSquared) were able to differentiate two patient groups (*P* = 0.02–0.04) with good AUC, sensitivity, and specificity ranging from 0.76 to 0.81, 71.4–78.6%, and 80–90%, respectively (Tables 2 and 3). Four GLCM features (Correlation, Imc1, Imc2, and MCC) significantly differentiated two groups (*P* = 0.004–0.04) showing good AUC, sensitivity, and specificity ranging from 0.73 to 0.86, from 42.9 to 78.6%, and from 80 to 100%, respectively (Tables 2 and 3) (Fig. 3). Between GLSZM features only one (LargeAreaLowGrayLevelEmphasis) significantly differentiated responders and non-responders (*P* < 0.0001), but no significant result was obtained in ROC curve analysis (*P* = 0.523) showing poor AUC, sensitivity, and specificity (0.58, 35.7, and 100%, respectively) (Tables 2 and 3). Among radiomic features extracted from portal phase, only one Shape feature (SurfaceVolumeRatio) resulted to be statistically significantly different between responders and non-responders (*P* = 0.04) showing good AUC, sensitivity, and specificity of 0.74, 71.4, and 70%, respectively (Tables 2 and 3). All significant radiomic features obtained, for both arterial and portal phase, were also tested in the external synthetic cohort and all features were confirmed to be significantly different with P value ranging from 0.002 to < 0.001 and AUC from 0.58 to 0.90 (Tables 2, 3, and 4).

**Table 2:** Significant radiomic features resulted in comparison between responders (PFS ≤ 11) and non-responders (PFS > 11) NETs in internal and external cohorts

Radiomic features	PFS ≤ 11	PFS > 11	P Internal cohort	Bonferroni correction	PExternal cohort	Bonferroni correction
Arterial phase	Mean ± SD	Mean ± SD				
First Order_10Percentile	49.93 ± 13.19	38 ± 12.07	0.03	–	0.002	–
First Order_Mean	70.12 ± 12.79	59.76 ± 9.85	0.04	–	0.002	–
First Order_Median	70 ± 12.67	59.9 ± 9.62	0.04	–	0.002	–
First Order_ RootMeanSquared	76.82 ± 15.41	63.27 ± 9.40	0.02	–	0.0001	–
GLCM_Correlation	0.29 ± 0.21	0.17 ± 0.05	0.001	–	< 0.0001	** < 0.0001**
GLCM_Imc1	− 0.07 ± 0.16	− 0.03 ± 0.01	0.004	–	< 0.0001	** < 0.0001**
GLCM_Imc2	0.32 ± 0.23	0.21 ± 0.06	0.01	–	< 0.0001	** < 0.0001**
GLCM_MCC	0.42 ± 0.3	0.28 ± 0.07	0.04	–	< 0.0001	** < 0.0001**
GLSZM_LargeAreaLowGrayLevelEmphasis	191,313.61 ± 228,501.47	317,486.48 ± 588,025.78	< 0.0001	** < 0.0001**	0.04	–
*Portal phase*						
Shape_SurfaceVolumeRatio	0.07 ± 0.01	0.06 ± 0.008	0.04	–	< 0.0001	** < 0.0001**

Bold values denote statistical significance

*PFS* Progression-free survival, *SD* Standard deviation, *GLCM* Gray-level co-occurrence matrix, *GLSZM* Gray-level size zone matrix

**Table 3:** Performance of radiomic parameters in comparison between responders and non-responders tested by using receiver operating characteristic (ROC) curve in internal cohort

Radiomic features	PFS $\le$ 11 vs PFS > 11				
Arterial phase	Sensitivity	Specificity	AUC	Criterion	*P*
First Order_10Percentile	78%	80%	0.77	> 44	**0.01**
First Order_Mean	71.4%	90%	0.76	> 65.6	**0.02**
First Order_Median	71.4%	90%	0.76	> 66	**0.01**
First Order_ RootMeanSquared	78.6%	80%	0.81	> 66.9	**0.001**
GLCM_Correlation	78.6%	80%	0.86	> 0.21	** < 0.0001**
GLCM_Imc1	78.6%	80%	0.84	$\le$-0.04	** < 0.0001**
GLCM_Imc2	42.9%	100%	0.76	> 0.29	**0.01**
GLCM_MCC	50%	100%	0.73	> 0.42	**0.03**
GLSZM_LargeAreaLowGrayLevelEmphasis	35.7%	100%	0.58	$\le$ 22,113.22	0.52
*Portal phase*					
Shape_SurfaceVolumeRatio	71.4%	70%	0.74	> 0.06	**0.01**

Bold values denote statistical significance

^*^PFS Progression-free survival, *AUC* Area under the curve, *GLCM* Gray-level co-occurrence matrix, *GLSZM* Gray-level size zone matrix

**Table 4:** External validation of radiomic parameters performance in comparison between responders and non-responders by using receiver operating characteristic (ROC) curve

Radiomic features	PFS $\le$ 11 vs PFS > 11				
*Arterial phase*	Sensitivity	Specificity	AUC	Criterion	*P*
First Order_10Percentile	59%	78%	0.66	> 49.9	**0.0001**
First Order_Mean	59%	79%	0.63	> 65.5	**0.002**
First Order_Median	63%	74%	0.63	> 64.9	**0.002**
First Order_ RootMeanSquared	66%	74%	0.67	> 68.8	** < 0.0001**
GLCM_Correlation	81%	84%	0.90	> 0.21	** < 0.0001**
GLCM_Imc1	74%	85%	0.84	≤ 0.037	** < 0.0001**
GLCM_Imc2	45%	97%	0.74	> 0.28	** < 0.0001**
GLCM_MCC	68%	97%	0.82	> 0.35	** < 0.0001**
GLSZM_LargeAreaLowGrayLevelEmphasis	25%	95%	0.58	≤ 32,331.22	**0.04**
*Portal phase*					
Shape_SurfaceVolumeRatio	39%	100%	0.69	> 0.07	** < 0.0001**

Bold values denote statistical significance

^*^PFS Progression-free survival, *AUC* Area under the curve, *GLCM* Gray-level co-occurrence matrix, *GLSZM* Gray-level size zone matrix

In the analysis of interobserver variability, three features (Median, Correlation, and Imc1) resulted stable, with 0.77 ≤ ICC < 0.83, and one excellent (LargeAreaLowGrayLevelEmphasis) with ICC = 0.92. All these figures were extracted from arterial phase.

### Survival analysis

**Fig. 4:**
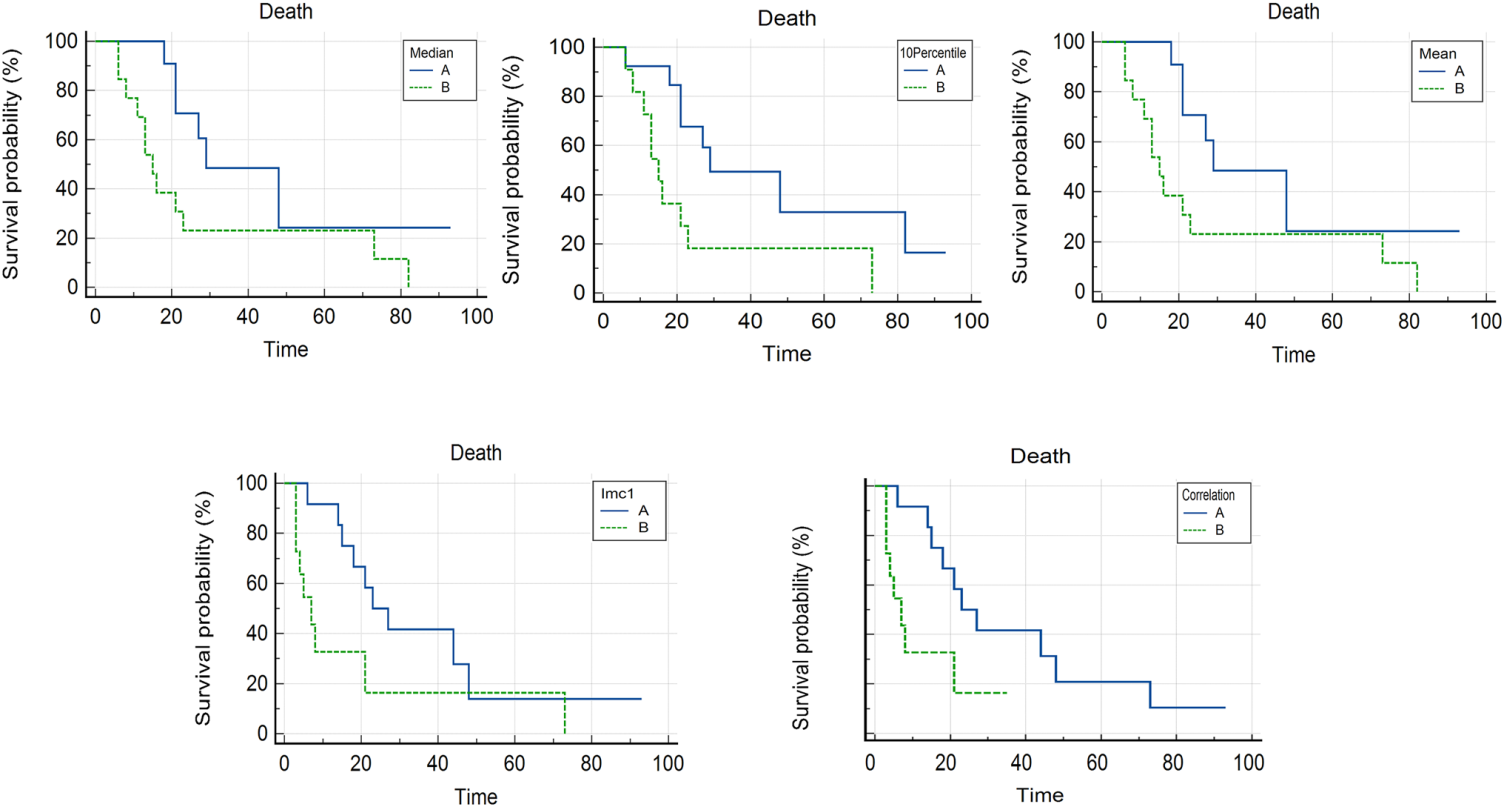
Kaplan–Meyer curves of radiomic features resulted to be significantly correlated with death (*P* < 0.05). Time was expressed in months

Kaplan–Meyer analysis showed that five features extracted from late arterial phase, if dichotomized at the best threshold according to PFS, resulted to be statistically correlated with death before starting Everolimus: 10Percentile (First Order) had cutoff of 44 with *P* = 0.01, Mean (GLMC) had cutoff of 65.6 with *P* = 0.02, Median (GLMC) had cutoff 66 with *P* = 0.02, Correlation (GLMC) had cutoff 0.21 with *P* = 0.02, and Imc1 (GLMC) had cutoff – 0.04 with *P* = 0.05 (Fig. 4).

### Univariate and multivariate analyses

Univariable logistic regression analysis was performed for assessing the correlation between clinical and radiomic features with death. Univariate analysis showed that no clinical parameters were significantly associated with death: age (*P* = 0.50), sex (*P* = 0.54), tumor grading (*P* = 0.29), Ki67 (*P* = 0.20), pancreatic and ileal primary (*P* = 0.57 and *P* = 0.41, respectively). Among radiomic features, extracted from arterial phase, two GLSZM features resulted to be predictors of death: GrayLevelVariance (OR 1.61; 95%CI, 0.65 to 3.94; *P* = 0.05); ZonePercentage (OR, 3.59; 95%CI, 2.06 to 62,529,763.18; *P* = 0.04). While, one GLSZM feature resulted to be inversely correlated with death: GrayLevelNonUniformity, (OR, 0.99; 95%CI, 0.98 to 1; *P* = 0.04), and three GLSZM features resulted to be irrelevant: LargeAreaEmphasis (OR, 1; 95%CI, 1 to 1; *P* = 0.008), LargeAreaLowGrayLevelEmphasis (OR, 1; 95%CI, 1–1; *P* = 0.02), and ZoneVariance (OR, 1; 95%CI, 1 to 1; *P* = 0.008) (Table 5). The remanent radiomic features resulted to be not statistically correlated with death.

**Table 5:** Univariate and multivariate logistic regression to test the correlation between radiomics and death

	Univariate analysis			Radiomic model		
Variable	OR(95%CI)	P	Coefficient	OR(95%CI)	P	Coefficient
Age	–	0.50	–	–	–	–
Sex (F = 0)	–	0.54	–	–	–	–
Grading	–	0.29	–	–	–	–
Ki67	–	0.20	–	–	–	–
Pancreatic	–	0.57	–	–	–	–
Ileal	–	0.41	–	–	–	–
GLSZM_GrayLevelVariance	1.61 (0.65–3.94)	**0.05**	0.48	1.72 (1.04–2.83)	**0.03**	0.54
GLSZM_ZonePercentage	3.59 (2.06–62,529,763.18)	**0.04**	– 146.08	9.76 (1.56–6.12)	**0.02**	– 368.44
GLSZM_GrayLevelNonUniformity	0.99 (0.98–1)	**0.04**	-0.006	–	–	–
GLSZM_LargeAreaEmphasis	1 (1–1)	**0.008**	0.0001	–	–	–
GLSZM_LargeAreaLowGrayLevelEmphasis	1 (1–1)	**0.02**	0.0001	–	–	–
GLSZM_ZoneVariance	1 (1–1)	**0.008**	0.0001	–	–	–

Bold values denote statistical significance

*OR Odds ratio, *GLSZM* Gray-level size zone matrix

Multivariate radiomic model was built by including radiomic features resulted significantly correlated with death from univariate analysis. The best radiomic parameter, predictor of death, was GrayLevelVariance (OR, 1.72;95%CI, 1.04–2.83; *P* = 0.03). Prognostic performance of multivariable radiomic model showed good AUC of 0.87 (95% CI, 0.67–0.97), sensitivity of 100%, and specificity of 66.7% (Table 5).

## Discussion

In this study, we tested radiomics as an imaging tool with the expectancy to be a predictor of progression-free survival and death in patients affected by metastatic progressive NENs, who can benefit of Everolimus. We had focused the study on PFS as a clinical endpoint according to the consistent evidence which described OS as a reductive outcome biomarker in the assessment of NENs [24]. Furthermore, we considered to divide the initial population according to PFS following the results obtained in the trails RADIANT 3 and 4 [18, 19]. Our results showed that ten radiomic features, extracted from naïve CT scans, were able to differentiate responders from non-responders (*P* < 0.05) with good performance (AUC 0.73–0.88), these data were also validated through an external validation cohort achieving some relevant data in terms of performance. In the survival analysis, five radiomic parameters showed to be significantly correlated with death in survival analysis. Furthermore, interobserver variability was also tested, in the arterial phase three features resulted stable and one excellent, this latter was the same to be significant after applying Bonferroni correction. In univariate analysis, GrayLevelVariance and Zone Percentage (GLSZM features) resulted to be correlated with death. Only one GLSZM feature, GrayLevelNonUniformity, resulted inversely correlated with death having a value of OR extremely closed to 1 (0.99), then with weak consistency and that should be confirmed in a future second step of analysis. Multivariate analysis enabled to reach a promising radiomic model in death prediction reaching an AUC of 0.87, sensitivity of 100%, and specificity of 66.7%. An unexpected result was that no clinical data showed to be significantly linked to death, then patient outcome was not predictable by analyzing only medical evidence. Among clinical parameters, Ki67 is usually considered a key outcome biomarker, consistently correlated with tumor aggressiveness and patient prognosis [25]. Then, these nonsignificant results could be justified considering the heterogeneous population, especially in high number of G2 in comparison with G1 and G3, and small sample size.

Radiomics has been emerging in cancer imaging, representing the future imaging landscape, having the chance to become a supporting tool for the clinicians in the structured management and workup of oncologic patients [6, 26]. Recently, radiomic approach has been extensively investigated in oncology and consistent results were demonstrated in differential diagnosis, prediction prognosis, and response to therapy in several cancers [6, 7]. Pretreatment patient risk stratification could be useful for clinicians to have a strength approach with patients with high risk of progression before starting target therapy. Focusing on NETs, several promising results have been already reached in differentiating well- from poor-differentiated NETs, in predicting tumor grading, and in differential diagnosis between pancreatic NETs and adenocarcinomas [11–15]. In particular, Bian Y. et al. [13] built a CT-based radiomic score to assess tumor grading in nonfunctioning pancreatic NETs with the aim to distinguish G1 from G2. They analyzed 102 CT scans of pancreatic NETs patients and multivariate logistic regression analysis was performed to test the correlation between radiomics and tumor grading; results demonstrated that radiomic score achieved good performance (AUC 0.86; sensitivity 94%, and specificity 63.5). Similarly, Gu et al. [14] in a multicentric study enrolled 138 patients with pancreatic NETs with the goal to test radiomics in preoperative prediction of tumor grading. They extracted 853 features from CT scans, analyzing both arterial and portal phases, and performed a nomogram, based on clinical and selected radiomic features, able to discriminate grade 1 from grade 2/3 pancreatic NENs with AUC of 0.974 and 0.902 in training and validation cohort, respectively. These studies confirmed the potential role of radiomics in management of NETs, especially to identify NETs with poor aggressiveness (G1 vs G2/3). Their results reinforced our data, which highlighted the promising role of radiomics to stratify patients according to tumor aggressiveness and the ability in distinguishing responders from non-responders NETs. Moreover, radiomics was also tested in differential diagnosis between atypical pancreatic NENs and pancreatic adenocarcinomas by He M. et al. [15], they developed three different models and radiomic outperformed clinic-radiological model (AUC 0.884 vs 0.775). Also, these results enhanced the strengths of radiomics in identifying more aggressive tumors, especially in case of atypical appearance of pancreatic NETs during imaging workup. We also showed that radiomics could outline tumor profile before starting target therapy, by highlighting microarchitecture and heterogeneity, then identifying patients who can be treated with Everolimus with great benefits. Then, radiomic approach could be considered in case of advanced NETs eligible to be treated with Everolimus to recognize potential good responders.

To date, some studies tested radiomics as a noninvasive biomarker to assess outcome in patients affected by NENs on preoperative medical images with the goal to provide an objective support to identify patients with more aggressive disease [10, 27–29]. In the future, radiomics could be also investigated and considered as noninvasive tool to support or replace re-biopsy in case of progressive disease, when the clinicians need revaluation of tumor aggressiveness [30]. One of the major studies was performed by the group of Song [27], which proposed a deep learning radiomic approach on preoperative CT scans to predict the recurrence risk in patients affected by pancreatic NENs treated with radical surgery. Their radiomic model, built on arterial phase, resulted promising to assess the risk of recurrence with AUC of 0.80. Also, texture analysis yielded some consistent results as prognostic biomarker when applied to baseline ^68^ Ga PET–CT scans in patients affected by metastatic NETs, in fact tumor textural heterogeneity resulted to be correlated with shorter PFS [28]. As well, Mapelli et al. [29] investigated radiomics performance in preoperative risk patient stratification by analyzing baseline FDG and ^68^ Ga PET/CT scans in PNETs patients eligible to surgery. These studies showed the potentiality of radiomics in NETs workflow, in particular quantitative imaging could cover the main gaps existing in patient risk stratification. Our data reinforced the idea that radiomics could have a future role in prediction prognosis, by providing a quantitative noninvasive tool to the clinicians. Our radiomic model reached good performance to predict death, especially two radiomic features showed direct correlation with death but no clinical data were significant. Concerning the lack of significant correlation between clinical parameters and death, we are in accordance with previous literature results regarding the difficulty of prediction prognosis only by using medical data in patients affected by advanced NENs [4, 31].

Furthermore, we want to focus the attention on our main results obtained from the analysis of arterial phase, this aspect highlights and confirms the key role of CT arterial phase in detection and characterization of NENs [32, 33]. In fact, some previous radiomic studies on NENs reported that their consistent data were obtained in the analysis of arterial phase, limiting the role of portal phase [10, 27]. This aspect was also confirmed in the qualitative study performed by Kim and colleagues [34], in which the evaluation of conventional CT findings on arterial phase achieved excellent data for predicting patient survival. Our data were in accordance with these literature evidence, in fact only one feature extracted from portal phase resulted significant. It was SurfaceVolumeRatio, a shape feature having the expectancy to reflect the compactness of whole, without any information about the heterogeneity. The remanent significant features, extracted from arterial phase, had the power to provide an objective evaluation of microstructural architecture, with different statistical levels (e.g., first and second order), considering pixel or voxel intensity and their relationship with the others [6].

In that context, radiomics might be central in starting workup of NEN patients, having the chance to be an objective and noninvasive imaging tool able to reflect tumor heterogeneity and to predict patient outcome by analyzing medical images before any therapies. The study has several limitations that should be overcome in the future second step. Firstly, the small sample size and heterogeneity of patients affected by metastatic NENs eligible to Everolimus treatment; secondly, the retrospective nature of the study; thirdly, the lack of external validation cohort; fourthly, no feature selection was performed. In the future, these drawbacks need to be overcome by expanding the starting population, performing a different analysis for each different primitive NET, also by selecting consistent feature and validating with external cohort.

To sum up, radiomics achieved good performance to differentiate patient responders from non-responders before starting target therapy. Moreover, radiomic model yielded consistent results in prediction patient outcome, while clinical data resulted to be not statistically correlated with prognosis. Radiomics could be considered as noninvasive imaging tool to stratify patients based on radiomic features reflecting tumor aggressiveness, before starting therapeutic workflow.

## Abbreviations

NENs: Neuroendocrine neoplasms
NETs: Neuroendocrine tumors
NECs: Neuroendocrine carcinomas
GEP-NETs: Gastroenteropancreatic Neuroendocrine tumors
PFS: Progression-free survival
OS: Overall survival
CM: Contrast medium
LBW: Lean body weight
GLSZM: Gray-level size zone matrix
NGTDM: Neighboring gray-tone difference Matrix
GLDM: Gray-level dependence matrix
GLCM: Gray-level co-occurrence matrix
GLRLM: Gray-level run length matrix
ROC: Receiver operating characteristic
ADASYN: Adaptive synthetic sampling approach
